# Activity of pemetrexed and high-dose gefitinib in an EGFR-mutated lung adenocarcinoma with brain and leptomeningeal metastasis after response to gefitinib

**DOI:** 10.1186/1477-7819-10-235

**Published:** 2012-11-07

**Authors:** Ying Yuan, Chunwen Tan, Modan Li, Hong Shen, Xuefeng Fang, Yinghong Hu, Shenglin Ma

**Affiliations:** 1 Department of Medical Oncology, Second Affiliated Hospital Zhejiang University College of Medicine, 88, Jiefang Road, Hangzhou, 310009, Zhejiang, China; 2 Neuroscience Care Unit, Second Affiliated Hospital Zhejiang University College of Medicine, 88, Jiefang Road, Hangzhou, 310009, Zhejiang, China; 3 Department of Oncology, Hangzhou First People’s Hospital, Hangzhou Cancer Hospital, 261 Huansha Road, Hangzhou, 310006, Zhejiang, China

**Keywords:** Non-small cell lung cancer, Pemetrexed, Epidermal growth factor receptor-tyrosine kinase inhibitors, Mutation

## Abstract

About 20% to 40% of patients with non-small cell lung cancer (NSCLC) will develop brain metastases during the natural course of their disease. The prognosis for such patients is very poor with limited survival. In addition to the standard whole brain radiation therapy (WBRT), some studies have shown that chemotherapy drugs and/or epidermal growth factor receptor-tyrosine kinase inhibitors (EGFR-TKI) can improve the outcome of these patients. Here, we report a stage IIIA patient who developed multiple brain metastases one year after operation. Oral gefitinib with concurrent WBRT were given as first-line therapy. Complete response and a 50-month progression-free survival (PFS) were obtained. Double dosage of gefitinib (500 mg per day) together with pemetrexed were given as the second-line therapy after the patient developed new brain lesions and leptomeningeal metastasis during the maintenance therapy of gefitinib. The PFS for the second-line therapy was six months. In total, the patient obtained an overall survival of 59 months since the first diagnosis of brain metastases. Mutational analysis showed a 15-nucleotide deletion and a missense mutation in exon 19 of the EGFR gene, and a missense mutation at codon 12 of the K-ras gene. These underlying genetic changes might partially explain the long-term survival of this patient after brain metastases when treated with concurrent or sequential therapies of EGFR-TKI, radiotherapy and chemotherapy.

## Background

The reported incidence of brain metastasis (BM) in patients with non-small cell lung cancer (NSCLC) ranges from 20% to 40%. Multiple cerebral lesions are associated with poor prognosis, and median overall survival from the time of diagnosis is about three to six months. Leptomeningeal metastasis (LM) of NSCLC is more fatal than BM, without treatment, median survival ranges from four to six weeks. The optimal treatment for such patients is controversial, and the use of a combination of radiotherapy, chemotherapy and EGFR-TKI remains unclear. The objective of the present case is to investigate the response to gefitinib and pemetrexed in such a patient.

## Case presentation

In June 2005, a 52-year-old man was admitted to our hospital after the detection of a mass in his right lung in a routine physical examination. He was a non-smoker and did not have any disease-related symptoms, such as cough, dyspnea or emptysis. Chest computerized tomography (CT) scan revealed a mass of 2.5 cm × 2.0 cm in size in the right upper lobe with ipsilateral, multiple bulky hilar and mediastinal lymph nodes. Abdominal CT scan, brain magnetic resonance imaging (MRI) and bone emission computerized tomography (ECT) scan showed no evidence of any distant metastasis. He was diagnosed with adenocarcinoma by a CT-guided percutaneous core needle biopsy. His clinical stage was cT1N2M0 (stage IIIA). Right upper lobectomy and systematic lymphadenectomy were performed after receiving informed consent. No adhesions to the visceral pleura were observed during the operation. Black swollen lymph nodes were found surrounding the carina, main bronchus, as well as in the mediastinum. Complete resection was achieved.

**Figure 1:**
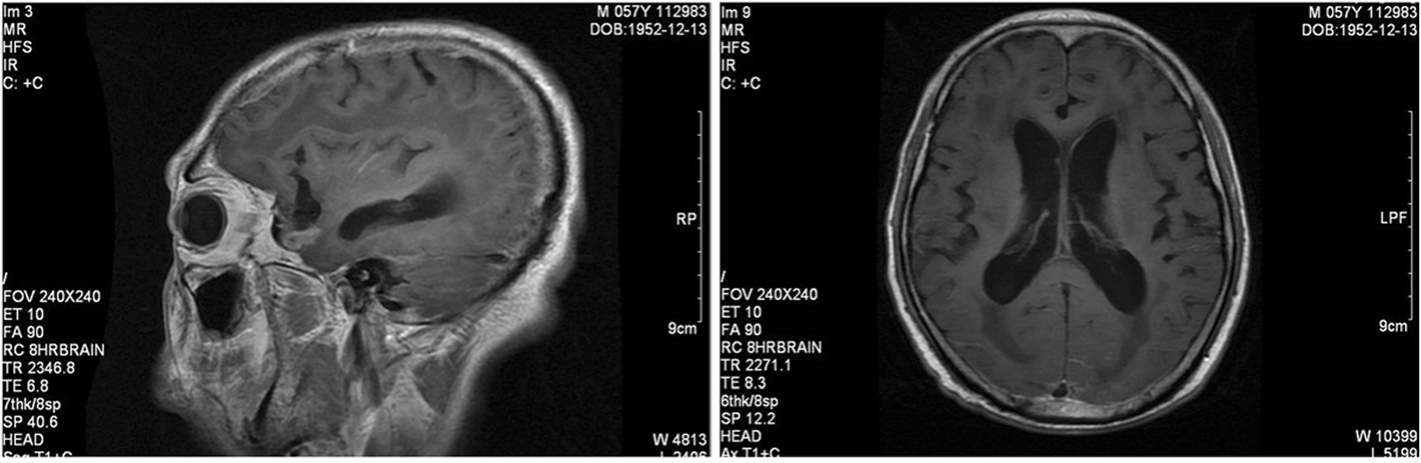
**Pathological findings showed irregular adenoid or papillary arrangement of cancer cells with invasion into the adjacent tissues.** Cells were rich in cytoplasma with obvious atypia and necrosis.

Pathologically, the primary lesion was 2.5 cm × 2.0 cm in size in the right upper lobe. The final pathological report showed adenocarcinoma with violations of the vascular supply, negative bronchial resection margins, and metastasis to hilar and mediastinal lymph nodes; 23/47 lymph nodes were positive (Figure 1). Immunohistochemical staining showed the tumor was negative for CgA, and positive for P53, PCNA, CK-L, CK-H, and NSE.

The patient received four cycles of adjuvant chemotherapy consisting of gemcitabine 1.0 g/m^2^ on day 1 and day 8, and carboplatin AUC 5 on day 1, repeated every 3 weeks. Sequential adjuvant radiotherapy with the dosage of 15 mV-X line DT 5000 cGy/25F irradiation on the mediastinal area was given.

About one year after operation, asymptomatic multiple cerebral metastases were detected during a routine follow-up, without any other recurrent extracranial lesions. As the patient refused systemic chemotherapy, we gave him gefitinib, 250 mg per day, and concurrent whole brain radiation therapy (WBRT) with the dosage of DT 4000 cGy/20F. Complete remission of the intracranial lesions was found one month after radiation. The patient continued to use gefitinib at 250 mg per day as maintenance therapy. A progression-free survival (PFS) of 50.0 months was obtained.

**Figure 2:**
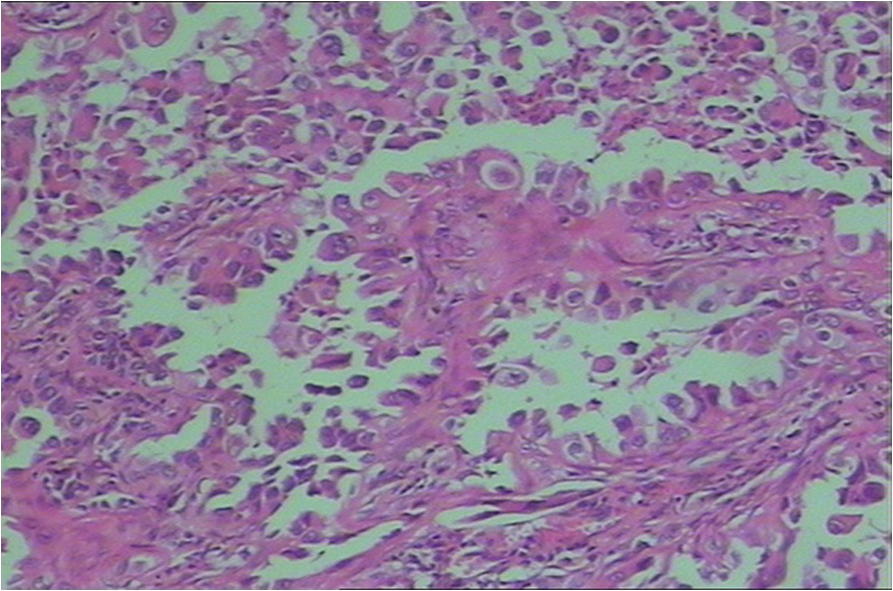
Magnetic resonance imaging (MRI) scan of the brain found dilated ventricles, diffuse white matter atrophy, and a metastatic mass about 1.5 cm in size in the right temporal lobe.

In August 2010, more than five years after the operation, the patient was readmitted to our hospital because of intense headache with nausea and vomiting. Emergent brain MRI found dilated ventricles, diffuse white matter atrophy, and also a metastatic mass about 1.5 cm in size in the right temporal lobe (Figure 2). Chest CT scan showed mild radiation pneumonitis without any evidence of tumor recurrence. The intracranial pressure measured by lumbar puncture was 220 mm H_2_O. Malignant cells were found in the cerebrospinal fluid. The patient was diagnosed with recurrent brain lesions and newly developed leptomeningeal metastases. A lumbar drainage catheter was lined to relieve intracranial hypertension symptoms. A double dosage of gefitinib of 250 mg twice a day combined with pemetrexed 500 mg/m^2^ on day 1 every 3 weeks were suggested. After six cycles of pemetrexed, no obvious changes were documented on the brain MRI and chest CT, but the symptoms of intracranial hypertension were completely relieved. The lumbar drainage tube was removed, all dehydration agents were stopped, and the performance score of the patient was improved from 2 to 1. Then, the patient stopped pemetrexed and went on with gefitinib single agent, 250 mg twice a day, obtaining a time to progression of 6.0 months.

At the end of February 2011, about two months after stopping pemetrexed, the patient had a recurrence of headache with nausea and vomiting, and the performance score deteriorated quickly to 4. The dosage of gefitinib was increased to 250 mg three times a day, however, no improvement was observed. The patient finally died of pulmonary infection, hernia and cachexia within three months.

The patient tolerated treatment well throughout the whole treatment process. No obvious adverse reactions were documented during the adjuvant chemotherapy and radiotherapy, the palliative whole brain radiation therapy, or the gefitinib treatment with a dosage of 250 mg per day. Grade 1 acne-like rash on the face and back, and itching of the lower limbs were observed during the gefitinib treatment with an increase in dosage to 500 mg or 750 mg per day. Grade 4 neutropenia and thrombocytopenia were documented in the first cycle of pemetrexed with the standard dosage of 500 mg/m^2^. With a 20% reduction of pemetrexed dosage and prophylactic use of granulocyte colony-stimulating factor (G-CSF) and recombinant human thrombopoietin (TPO), the patient completed the following five cycles smoothly. Repeated grade 1 liver dysfunction was observed during the pemetrexed treatment.

**Figure 3:**
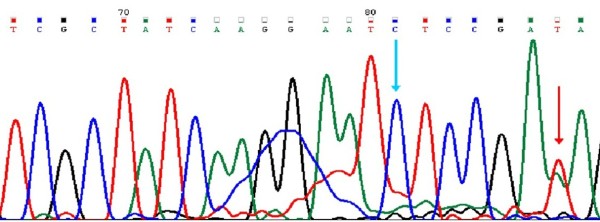
**Mutational analysis of EGFR gene by direct sequencing.** A deletion of 15 nucleotides in exon 19 of the EGFR gene (2240-2254del15; L747-S752del5) is indicated by a blue arrow. A point mutation in exon 19 of the EGFR gene (A2262T; K754I) is indicated by a red arrow.

**Figure 4:**
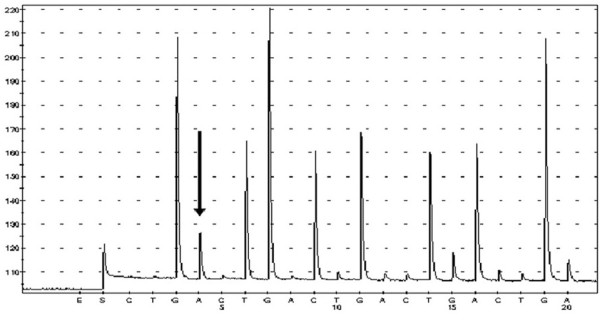
Mutational analysis of K-ras gene by pyrosequencing: a point mutation (arrow) in codon 12 (GGT→GAT; G12D) was detected.

The mutation analysis of related genes was carried out in 2010 when the patient was diagnosed with recurrent brain lesions and leptomeningeal metastases. Genomic DNA was isolated from the tumor specimen obtained from the operation five years earlier, after receiving informed consent. The direct sequencing after PCR amplification or pyrosequencing analysis were performed to detect mutations in exons 19, 20 and 21 of the EGFR gene, codon 12 and 13 of the K-ras gene, and codon 600 of the BRAF gene. A deletion of 15 nucleotides (2240-2254del15; L747-S752del5) and a point mutation (A2262T; K754I) in exon 19 of EGFR gene were detected (Figure 3). Both exons 20 and 21 were wild type. A point mutation of codon 12 (GGT→GAT; G12D) of the K-ras gene was also identified, which led to an amino acid change from glycine to aspartic acid (Figure 4). The codon 600 of the BRAF gene was wild type.

## Discussion

The prognosis for NSCLC patients with BM is poor, with a natural history of about three to six months and a 10% one-year survival rate [1]. Whole brain radiation therapy (WBRT) is the standard treatment for BM patients. Small molecules such as epidermal growth factor receptor-tyrosine kinase inhibitors (EGFR-TKIs) like erlotinib, gefitinib and lapatinib have been demonstrated to have the ability to cross the blood–brain barrier (BBB). The use of chemotherapy for the treatment of BM has been limited because of a presumed lack of effectiveness due to the BBB. However, in recent years, some studies found that WBRT combined with systemic chemotherapy or EGFR-TKIs could further improve the outcome [2-4]. Kim *et al*. [3] reported an improvement of median overall survival from 19.0 weeks to 58.1 weeks in patients treated with WBRT and platinum-based chemotherapy as compared to those with WBRT only. A response rate of 81% and a disease control rate of 95% were reported by Ma *et al*. [4] in their 21 NSCLC patients with BM treated with concomitant WBRT and gefitinib. The median PFS and median overall survival (OS) were 10.0 months and 13.0 months, respectively. However, in cases where the patients had tumor progression of intracranial lesions during or after completion of radiotherapy, no standard and effective treatment could be recommended. Chemotherapy or targeted agents could be used as an alternative.

The incidence of pulmonary adenocarcinoma is increasing and it has become the leading subtype of lung cancer according to recent statistics. Currently, pemetrexed constitutes a commonly used standard second-line treatment and first-line treatment option in patients with metastatic nonsquamous cell lung cancer. Some studies had shown the activity of pemetrexed for the treatment of brain metastases with low toxicity [5-7]. Bearz *et al*. [6] reported an overall clinical benefit (stable disease (SD) and partial response (PR)) and a cerebral benefit of 63% and 68% respectively in 22 NSCLC patients with brain metastases treated with pemetrexed plus cisplatin. Patients were irradiation-naive or with clear radiological evidence of cerebral progression after brain radiotherapy and before pemetrexed treatment. Another study by Barlesi *et al*. [7] also demonstrated a pemetrexed-cisplatin regimen was effective and well-tolerated for 43 NSCLC patients with cerebral metastasis. Cerebral, extracerebral and overall response rates were 41.9%, 34.9% and 34.9% respectively. The disease control rate was 72.1%. The median OS, median PFS and median time to cerebral progression were 7.4 months, 4.0 months and 5.7 months. One patient even had complete cerebral remission (1/43, 2.3%). The possible mechanisms of pemetrexed therapeutic activity in brain metastasis might be as follows: (1) Molecular studies demonstrated that P-glycoprotein was a major constituent of the BBB acting as an effective efflux transporter protein. In contrast to primary cerebral malignomas, brain metastases seemed to express less P-glycoprotein leading to increased drug permeability of the BBB. P-glycoprotein activity is usually measured in the tumor cells and does not change the BBB permeability of drugs. BBB is dependent on vascular not tumor cells [8]. Therefore, lower levels of P-glycoprotein might contribute to the effect of pemetrexed on NSCLC brain metastasis. (2) Auxiliary use of dexamethasone and mannitol during the treatment might increase BBB permeability and cerebral concentrations of drugs. (3) Pemetrexed might upregulate the local bradykinin B2 receptors and affects the secretion of bradykinin to play a therapeutic role, although the mechanism is unclear [9].

In addition to chemotherapy drugs, oral EGFR-TKIs had shown remarkable efficacy in NSCLC patients with brain metastases [10-12]. The EGFR-TKIs have excellent cell penetration of the BBB because of its chemical structure and low molecular weight. In 2003, Cappuzzo *et al*. [10] first reported one complete and three partial responses after three months of single gefitinib treatment in four NSCLC patients with pretreated brain metastasis. Thereafter, more studies presented further evidence of gefitinib efficacy in patients with brain and leptomeningeal metastasis [13,14]. However, it was reported that 57% patients had central nervous system (CNS) relapse during the gefitinib therapy, while there was no progression in extracerebral lesions. No standard treatment could be recommended for such patients. Jackman *et al*. [15] reported erlotinib-refractory leptomeningeal metastases from lung adenocarcinoma responds to high-dose gefitinib treatment, and highlighted that CNS metastases did not always harbor resistance mutations and suggested they retained erlotinib/gefitinib sensitivity if therapeutic drug concentrations were achieved. It was reported by Clarke *et al*. [16] that in patients developing meningeal metastasis during TKIs (erlotinib) treatment, the intermittent or pulsatile high-dose administration (1000 to 1500 mg/week) achieved a higher cerebrospinal fluid concentration than standard dosing, and successfully controlled LM in this patient.

Our patient developed multiple brain metastases one year after the operation. Whole brain radiation therapy combined with oral gefitinib was the first-line therapy, and the patient had great benefit with a PFS of more than four years. Double dosage of gefitinib (500 mg per day) combined with pemetrexed was the second-line therapy after the patient developed new brain lesions and leptomeningeal metastases, despite adequate control of the lung cancer outside the CNS. The PFS for the second-line treatment of gefitinib and pemetrexed was 6.0 months. The patient had a total OS of about five years after the first diagnosis of brain metastasis, which was extremely good as compared with survival data reported in the literature. The gefitinib treatment was effective for more than four years for first-line and effective again for six months with a double dosage together with pemetrexed for second-line treatment, which might be related to the sensitivity mutations detected in exon 19 of the EGFR gene, a deletion mutation of 15 nucleotides and a missense mutation downstream [17,18]. Co-existence of EGFR and K-ras mutations was seldom reported, only accounting for about 5% of EGFR-mutated patients [19]. Retrospective investigations of K-ras mutational status implied that K-ras mutation might be a negative predictor of response and a mechanism of *de novo* resistance to EGFR-TKI, even in patients with EGFR mutations [19-21]. However, small sample sizes as a result of low prevalence of K-ras mutations and the low rate of tumor sample collection have limited the strength of these analyses. And in this case, the role of the missense mutation detected at codon 12 of the K-ras gene remains to be elucidated. To a certain extent, it might contribute to the limited PFS and OS after the patient developed new brain lesions and leptomeningeal metastasis. Although, both gefitinib and pemetrexed had shown some therapeutic effects in patients with brain or leptomeningeal NSCLC metastasis, the effect of the combination of these two drugs remained uncertain. It was even hard to distinguish whether the patient benefited from gefitinib or pemetrexed. Learning from this case, the combination therapy of pemetrexed and gefitinib might have a better prospect. Prospective studies with a large sample should be carried out to further clarify the efficiency and underlying mechanisms.

## Conclusion

In conclusion, the concurrent or sequential use of radiotherapy, chemotherapy and EGFR-TKI might have a better prospect in EGFR-mutated NSCLC patients with brain metastasis and/or leptomeningeal metastasis.

## Consent

Written informed consent was obtained from the patient for publication of this case report and accompanying images. A copy of the written consent is available for review by the Editor-in-Chief of this journal.

## Abbreviations

BBB: blood–brain barrier; BM: brain metastasis; CNS: central nervous system; (E)CT: emission computerized tomography; EGFR-TKI: epidermal growth factor receptor-tyrosine kinase inhibitor; LM: leptomeningeal metastasis; MRI: magnetic resonance imaging; NSCLC: non-small cell lung cancer; OS: overall survival; PCR: polymerase chain reaction; PFS: progression-free survival; WBRT: whole brain radiation therapy.

## Competing interests

The authors declare that they have no competing interests.

## Authors’ contributions

YY wrote the manuscript. CWT, MDL and HS participated in the clinical management of the patient. XFF and YHH carried out the pathological examination and gene analysis. SLM was involved in the final editing. All authors approved the final manuscript.
